# Lessons learned on teaching a global audience with massive open online courses (MOOCs) on health impacts of climate change: a commentary

**DOI:** 10.1186/s12992-019-0494-6

**Published:** 2019-08-22

**Authors:** Sandra Barteit, Ali Sié, Maurice Yé, Anneliese Depoux, Valérie R. Louis, Rainer Sauerborn

**Affiliations:** 1 Heidelberg Institute of Global Health, University Hospital Heidelberg, University Heidelberg, Im Neuenheimer Feld 130.3, 69120 Heidelberg, Germany; 2 Centre de Recherche en Santé de Nouna, Nouna, Burkina Faso; 3 Centre Virchow-Villermé for Public Health Paris-Berlin, Université Sorbonne Paris Cité, Paris, France; 4 Groupe de Recherches Interdisciplinaires sur les Processus d’Information et de Communication (GRIPIC), Université Paris Sorbonne, Paris, France; 5 Harvard T.H. Chan School of Public Health, Boston, USA

**Keywords:** Health, Climate change, Global health, Global education, Global audience, Capacity building, Massive open online course, MOOC

## Abstract

**Background:**

The adverse health impacts of climate change are increasing on a global level. However, knowledge about climate change and health is still unavailable to many global citizens, in particular on adaptation measures and co-benefits of health mitigation. Educational technologies, such as massive open online courses (MOOCs), may have a high potential for providing access to information about climate change links to health for a global audience.

**Main body:**

We developed three MOOCs addressing the link between climate change and health to take advantage of the methodology’s broad reach and accelerate knowledge dissemination on the nexus of climate change and health. The primary objective was to translate an existing face-to-face short course that only reached a few participants on climate change and health into globally accessible learning opportunities. In the following, we share and comment on our lessons learned with the three MOOCs, with a focus on global teaching in the realm of climate change and health.

**Conclusions:**

Overall, the three MOOCs attracted a global audience with diverse educational backgrounds, and a large number of participants from low-income countries. Our experience highlights that MOOCs may play a part in global capacity building, potentially for other health-related topics as well, as we have found that our MOOCs have attracted participants within low-resource contexts. MOOCs may be an effective method for teaching and training global students on health topics, in this case on the complex links and dynamics between climate change and health and may further act as an enabler for equitable access to quality education.

## Background

The topic of climate change and its impact on health is increasingly present in public and academic discourse. Yet, research funding still lags [1] behind other sectors affected by climate change, e.g., agriculture or marine systems [2, 3]. Moreover, expertise to conduct research in climate change and health is unequally distributed between the Global North and South [4]. There is an urgent need for more professional training to stimulate engagement of climate change on a global level and to expand global engagement with and understanding of climate change and health, as well as research efforts in this topic area [5]. To this end, massive open online courses (MOOCs) show promise for: (i) quickly disseminating general knowledge on the topic for global citizens and (ii) adapting knowledge to specific contexts, e.g. high-level policymakers or particular settings in sub-Saharan Africa [6]. MOOCs have been taken up by major universities worldwide and have been heralded for revolutionizing global education since MOOCs are “open–access online courses that allow for unlimited participation” [7]. With more than 150 million students in education worldwide [8], MOOCs have shown great potential in their extensive global reach.

To harness MOOCs’ advantages for climate change and health education, we developed three MOOCs on this topic:

(i) the MOOC “*Climate Change and Health”* (MOOC-GP) on the learning platform called *iversity* [9] targeted a general audience introducing the nature of the health impact of climate change worldwide, best practices of adaptation and mitigation strategies and health co-benefit promotion as drivers for climate policy
(ii) MOOC “*Changement climatique et santé dans le contexte Africain”* (MOOC-AFR) on the FUN-MOOC learning platform [10] was developed for a general Francophone audience with a focus on climate change and health in sub-Saharan Africa and in cooperation with the *Centre de Recherche en Santé* (CRSN) in Nouna, Burkina Faso
(iii) MOOC “*Climate Change and Health for Policy-Makers”* (MOOC-PM) served as a brief for policy-makers to teach the essentials of climate systems and present the current debate on mitigation and adaptation policies.

The primary objectives were to (i) translate an existing face-to-face short course that only reached a few participants on climate change and health into globally accessible learning opportunities and to (ii) test the educational method of MOOCs for health-related topics. In the following, we want to share and discuss our experiences on strengths and shortcomings of MOOCs for global teaching in the realm of climate change and health.

### What are the advantages and shortcomings of MOOCs in climate change and health education?

Overall, there was no a priori goal of enrolment for the three MOOCs, except that the MOOC-PM was targeted to reach policy makers in particular. The MOOC-AFR was aimed at people from or working in francophone West Africa. The reach with over 7000 registered students (for more details, see [6]) was more than overwhelming - which was accompanied with a certain anxiety on how to handle the number of participant requests - which turned out fine in the end as the majority of interaction was based on peer-to-peer communication and peer support between participants. In an evaluation that we conducted (for more details, see [6]), we have found that the three MOOCs have reached people from diverse educational and geographical backgrounds, such as researchers and implementers in the field, decision-makers, and citizens from low-resource environments who are most vulnerable to climate change effects on health. Notably, the MOOC-AFR sparked high interest from African countries with many participants from low-resource countries, as compared to the other two MOOCs [6]. The focus of the MOOC-AFR was on the local environmental and socioeconomic context of Burkina Faso, and respective mitigation and adaptation strategies.

This African focus on the MOOC content was potentially an enabling factor to the high number of participants from low-resource countries - which is striking considering the constraints low-resource countries are faced with, such as low broadband penetration and comparatively high costs for Internet access through mobile data networks, as compared to average monthly income levels. Furthermore, the scarcity of courses on climate change and health especially with a focus on low-resource countries [11﻿] may have also been an enabling factor.

However, a gaping lack remains, regarding what the participants really learned from the respective MOOCs, especially of those who stopped the online courses early. The chain of evidence from the participation in the MOOC to the translation into real change and impact is difficult to uncover. Yet, this should not be a discouraging argument as developing new face-to-face curricula takes time and reaches only a few minds, particularly in the Global North. More studies are needed to ascertain the long-term effects and real penetration of knowledge gained by MOOC participants, as well as how to measure the effectiveness of a MOOC. As learners are global and have a diverse range of a priori knowledge and skills, many students may not be interested in completing a MOOC. Depending on the participants objective for taking part in the MOOC, some may rather specifically pick MOOC sections, as they see fit and need for themselves. A reason may be that the diversity of the learners may not follow traditional learning trajectories, that is reflected in evaluation measures such as course completion. This leads also to another question: How to best cater to such different learner types? One answer may be adaptive learning which allows adapting content to different learner types based on answers given throughout course quizzes or other student-based feedback, for example. However, each adaptive learning path has to be specifically developed which may require a certain time-investment from MOOC creators. A mean to ensure that participants have covered the course objectives may be via examination, such as officially verified exams, called proctored exams. Offering such exams for MOOCs, again, require financial and personnel resources. Insights on the effectiveness and benefits of adaptive learning also remain limited, as only few MOOCs have adopted this method. Evaluation of learning and acquiring knowledge and skills via MOOCs is complex and more research is needed to understand strengths and weaknesses of MOOCs to further guide best practices.

MOOCs shift access to education from a centralized and local, to a decentralized and global realm. The translation from a face-to-face course into a MOOC that typically consists of a collection of averagely 8-min long videos framed by exercises and collaborative activities involves time and effort. Some MOOC-platforms schedule about 1 year from the planning of a MOOC from scratch to the actual realization of the MOOC. Our MOOCs were developed in a shorter time frame as the face-to-face courses served as the base for the MOOC outline and contents, but the translation into a MOOC-format still involved considerable time and effort.

MOOCs offer a variety of formats such as teacher-centered videos (the case for our MOOCs), interview-based videos or micro-teaching – learning sessions that comprise only a few minutes each - that may be especially adequate for knowledge and skills transfer in a working environment where time is more constrained but still allows for short MOOC sessions. Another possibility that we explored was to blend MOOCs with face-to-face courses. To this end, participants from MOOCs were invited to one of our previously run presence courses. Within the face-to-face course we interwove video segments of the MOOC to transfer theory-based contents which were followed up by group work and other in-group activities to strengthen this knowledge. Informal feedback that we received of this blend of face-to-face with MOOC elements was quite positive by course participants and thus may constitute a potential cross-utilization of MOOCs for presence-courses, for example, to incorporate MOOCs as a fruitful add-on for Ph.D. programs. But again, more research would be needed in this area, also with regards to what constitutes a beneficial blend of MOOC segments and face-to-face interactions.

### Development of a MOOC

In total, 12 scientific experts from five countries designed the MOOC based on a face-to-face course at Heidelberg University. The endeavor to realize these three MOOCs was mainly driven by the efforts of a single senior faculty member (RS). A professional course and multimedia development team of the Centre Virchow-Villermé supported the design and implementation of the three MOOCs on the respective online learning platforms. They also actively supported participants in the MOOCs. The MOOC-GP and MOOC-PM were filmed in Europe in a small movie studio of the Centre Virchow-Villermé. The MOOC-AFR was filmed in Burkina Faso, and the majority of the experts that were teaching in the MOOC-AFR were Burkinabe themselves, as was the ownership and organization of this MOOC. The Centre Recherche en Santé Nouna in Burkina Faso coordinated and included lecturers from national universities and meteorological department of Burkina Faso. The MOOC-AFR was actively supported by French-speaking experts of the collaborating institute (CRSN) who helped answer discussion forum questions for 6 weeks (February – March 2017).

The MOOC-GP was actively supported for 5 weeks (February–March 2016), and included weekly live question-and-answer sessions and a discussion forum supported by experts who answered student questions.

The MOOC-PM had five chapters on why health is critical for climate negotiations and was actively supported for 5 weeks (from January–February 2016).

The MOOCs were translated from face-to-face courses resulting in readings and quizzes with mainly traditional instructional methods comprising lecture segments (with an average runtime of 9 minutes), potentially not engaging the participants actively enough. Collaborative elements were included in the MOOCs that followed instructional design principles, such as (i) problem-centered learning (real-world problems of climate change and health were discussed in video segments, such as health co-benefits of improving nutritional diets, of riding your bike, of reducing indoor air pollution), with (ii) application (through a real-life project) and (iii) integration of knowledge (tasks to discuss course topics in the discussion forum with fellow participants). More collaborative activities may engage participants even further in the topic area and could comprise of teamwork-based exercises and discussion forums, additional exchange spaces for collaboration like social networking or interest groups, as well as instructor feedback by the respective MOOC instructor and by peers.

Following the actively supported phase, all three MOOCs were still available on the respective learning platforms, as were the discussion boards for students to exchange information and discuss various topics. But further support to participants’ questions was no longer covered by course developers and involved experts. The MOOCs were still available online, however, no further strategy has yet been put in place on how to keep contents updated. Potentially, at one point the MOOCs are archived and materials may be used as the base for a new MOOC on the topic.

To lower the language barrier of participants’ to take part in the MOOCs subtitles were provided for the MOOC-GP and MOOC-PM in different languages (MOOC-GP: Arabic, Mandarin Chinese, English, French, Hindi, Indonesian, Portuguese, Russian and Spanish; MOOC-PM: Arabic, Chinese, English, German, Indonesian, Portuguese, Russian, Spanish). The subtitles were translated by native speakers, mostly doctoral and Master course students, who were familiar with the domain language of climate change and health (cf. [6]). For the translation, we made use of an open-source online platform which also allows for crowd-sourcing [12]. Such strategies, like offering subtitles, may further enable the reach and attractiveness of MOOCs for a global audience, but further research is needed to understand the effectiveness of subtitles.

To reach participants of different backgrounds, such as participants from outside the realm of the “usual suspects”, such as decision-makers, participants from low resource contexts and non-academic participants, we followed a “blended marketing” strategy, which combined online and “real-life” or offline marketing. This included reaching out in meetings or conferences or using the Universities’ alumni networks.

For example, the MOOC-PM was launched at the World Health Summit.

### Involved costs for a MOOCs

Beyond the effort of the senior faculty member, there was no institutional budget available for the development of the MOOCs. The technical support of the MOOC and its respective platform was provided by the Centre Virchow-Villermé based on a cooperation between participating institutions. For these MOOCs, a return of (financial) investment was not taken into account, as the primary objective was to translate the face-to-face course into a globally accessible MOOC format, with open access to quality knowledge of climate change and health. As the three MOOCs have shown, there is a general global interest in the topic of health influenced by climate change (cf. [6]). However, upfront and continuous investment of financial and human resources for MOOCs should best be considered and taken up by Universities and institutes in their educational strategy and budget to make MOOCs sustainable. Furthermore, for MOOCs to reach the status of a valid educational tool to become a part of an overall University teaching approach, teaching in a MOOC should be recognized as part of the official teaching assignment of faculty members. Such a recognition could further strengthen MOOCs as a teaching method.

The primary objective of MOOCs, as their name proclaims (open), is to make knowledge globally available to anyone. Yet, to sustainably support the MOOCs in the long run, the active support from Universities and institutes is vital.

With regards to the financial requirements, a further consideration may be to follow business models, as they are already in place from major universities that offer full degree-programs on a MOOC-basis. Such revenue models may support enrollment numbers and revenue for smaller universities and institutes [13]. However, MOOCs should remain open to anyone in their full content. A model that currently is followed is to charge for degrees or verified credit points but keep MOOC contents open to anyone for auditing. Potentially, scholarships or exemptions for people from a low-resource context could be introduced for MOOC-based degrees or verified credit points. This way MOOCs may, firstly, enable global students to access quality knowledge for free or a small fee which may translate into outcomes like changes in day-to-day behavior or in better skilled workforce. Secondly, MOOCs may be an income generator for institutions. Thirdly, MOOCs may increase the global visibility of the respective institution or University.

MOOCs can be costly regarding financial and human resources [14], but do not have to be. The content of the MOOC-AFR was developed with low-technological resources on location in Burkina Faso and included African experts in the field of climate change and health. The high participation rate (cf. [6]) indicates that MOOC productions may not necessarily need to put a high investment in high-quality video productions, but rather on the quality and relevance of content. Thus, MOOCs could foster global access to quality education. Especially low-resource countries, such as countries in Sub-Saharan Africa, have insufficient educational infrastructure to meet the educational need of their young population. Particularly with regards to critical shortages of human resources in health and healthcare that comprises limited numbers of medical teachers and limitations in physical infrastructures, such as classrooms [15].

**Table:** 

Key points of MOOCs for teaching health-related topics	
o MOOCs require upfront costs, time and effort	
o quality and relevance of contents of MOOCs seem to be of high relevance (more than quality of technical realization of MOOC contents)	
o MOOCs seem to be an adequate educational tool for making health-related topics available also for low-resource contexts	
o MOOCs may support reaching educational goals of low-resource contexts	
o MOOCs are versatile in their usage and composition (i.e. blended learning)	
o financial concept with the support of the University/institution should be in place for developing MOOCs, how to keep them up-to-date	
o MOOCs may be contributing to University/institute revenue and may increase global visibility	
o MOOCs seem an adequate tool for teaching about climate change and health in a global context	

## Conclusions

The three MOOCs have raised awareness among global citizens in the linkage of climate change and health, as they have reached researchers from diverse backgrounds and citizens from low-resource environments that are most vulnerable to climate change effects. Astonishingly, the francophone MOOC-AFR sparked high interest (see [6] for more details on evaluation results of the three MOOCs) in low-resource countries to learn more about climate change to pass it on to their community, families and professional environment. Costs for development and updating of the MOOC are to be accounted for, but even low-tech MOOCs that do not incorporate expensive “Hollywood”-effects may be successful teaching and learning tools if a pedagogical strategy is in place that incorporates an activating and stimulating environment beyond teacher-centered sessions. Based on our experience, MOOCs may play a part in global capacity building, even beyond the topic of climate change and its impact on health, and are a promising tool even in low-resource contexts. MOOCs may support creating awareness of health impacts of climate change, and act as an empowering instrument, even reaching the most vulnerable citizens.

## Data Availability

The datasets generated and/or analysed during the current study are not publicly available due to the terms of consent to which the participants agreed but are available from the corresponding author on reasonable request.
